# Linoleoyl-lysophosphatidylcholine drives non-inflammatory apoptosis in neutrophils via lipid rafts

**DOI:** 10.3389/fimmu.2026.1807766

**Published:** 2026-07-31

**Authors:** Priyanka Saminathan, Alicia Gibbons, Ian T. Mathews, Maija Corey, Ashmitaa Logandha Ramamoorthy Premlal, Mahati Rayadurgam, Namratha Nadig, Camille Fang, Neha Reddy, Mousa Vatanmakanian, Carolina Altbaum, Sonia Sharma

**Affiliations:** 1 Center for Autoimmunity and Inflammation, La Jolla Institute for Immunology, La Jolla, CA, United States; 2 Department of Medicine, University of California San Diego, La Jolla, CA, United States; 3 Laboratory for Inflammatory Immune Metabolism, Center for Integrative Medical Sciences, RIKEN, Yokohama, Japan

**Keywords:** apoptosis, inflammation, lipid rafts, lysophosphatidylcholine (LPC), NETosis, neutrophils, reactive oxygen species (ROS)

## Abstract

Lysophosphatidylcholines (LPCs) are among the most abundant circulating bioactive lipids, whose fatty acid compositions critically influence their immunomodulatory functions. Alterations in circulating LPC species have been implicated in inflammatory settings, including immune checkpoint blockade–associated immune-related adverse events (ICB-irAEs), yet how individual LPC species shape innate immune cell fate remains poorly defined. Here we investigated how unsaturated linoleoyl-LPC (LPC 18:2) and saturated palmitoyl-LPC (LPC 16:0) differentially regulate neutrophil survival and inflammatory responses through distinct cell-intrinsic mechanisms *in vitro*. In mature peripheral human neutrophils, LPC 18:2 markedly increased intracellular reactive oxygen species (ROS) generation and caspase-3/7 activation accompanied by mitochondrial membrane depolarization and cytochrome *c* release, consistent with activation of the intrinsic apoptotic pathway. In contrast, LPC 16:0 induced lower levels of ROS and elicited caspase-3/7 activation with a distinct dose-response profile to LPC 18:2. These were accompanied by lactate dehydrogenase (LDH) and high mobility group box 1 (HMGB1) release, as well as increased myeloperoxidase (MPO) activity, neutrophil elastase (NE) release, and citrullinated H3, indicating membrane rupture, NETosis-associated activity, and induction of a lytic form of cell death with damage-associated molecular pattern (DAMP) release. Bulk RNA sequencing revealed that LPC 16:0 strongly upregulated inflammatory and cytokine-associated gene expression programs, whereas LPC 18:2 treatment was largely transcriptionally quiescent. Disruption of lipid raft integrity through cholesterol depletion abolished LPC 18:2–induced intracellular ROS production and apoptosis, demonstrating a requirement for membrane organization in mediating these effects. Collectively, these findings identify LPC 18:2 as a non-inflammatory, lipid raft–dependent inducer of mitochondria-driven neutrophil apoptosis, whereas LPC 16:0 promotes inflammatory and lytic death pathways. These results highlight lipid saturation as a key determinant of neutrophil fate and immune tone, providing insight into how distinct LPC species may shape inflammation and tissue injury, including in the context of neutrophilic immunotherapy-associated toxicities.

## 1 Introduction

Immune checkpoint blockade (ICB) therapies targeting CTLA-4 and PD-1/PD-L1 have transformed cancer treatment, producing durable clinical responses across more than 20 solid tumor types, including melanoma, non-small cell lung cancer, and renal cell carcinoma. However, ICB therapy frequently leads to immune-related adverse events (ICB-irAEs), which are autoimmune-like toxicities resulting from off-tumor immune activation. These ICB-irAEs span a broad range of organ systems, including dermatologic, gastrointestinal, endocrine, hepatic, pulmonary, musculoskeletal, neurologic, cardiac, renal, and hematologic compartments, and can range from mild cutaneous reactions to life-threatening myocarditis (1). Severe grade III/IV ICB-irAEs significantly limit treatment tolerability and represent a major cause of therapy discontinuation (2). Current management strategies rely largely on systemic corticosteroids to control symptoms, but this approach induces broad immunosuppression and may compromise anti-tumor efficacy (3). Despite the clinical importance of ICB-irAEs, the cellular and molecular mechanisms that drive their development remain poorly understood, highlighting the need for targeted therapeutic strategies that restrain pathological immune responses while preserving anti-tumor immunity.

Neutrophils are increasingly recognized as dynamic and multifunctional regulators of ICB efficacy and toxicity (4, 5). In circulation, dysregulated neutrophil activation and expansion following ICB treatment can precipitate bursts of inflammatory effector activity that promote neutrophil tissue infiltration and immune-related damage (6–9). Consistent with this, an elevated neutrophil-to-lymphocyte ratio (NLR) is associated with worse overall and progression-free survival in patients receiving ICB and has been linked to the development of severe ICB-irAEs (10–13). Accordingly, modulation of neutrophil recruitment, function, and fate represents a promising strategy to improve ICB outcomes, as well as to mitigate other neutrophil-driven inflammatory pathologies (13, 14).

Whether neutrophils resolve or perpetuate inflammation is influenced by the mode of cell death. Neutrophil apoptosis induced by anti-inflammatory and pro-resolution lipid mediators has been shown to enhance inflammation resolution by enabling macrophage-mediated efferocytosis of the apoptotic bodies (15). Conversely, disturbed efferocytosis and cell death-related release of damage-associated molecular patterns (DAMPs) such as high mobility group box 1 (HMGB1) contribute to non-resolving tissue inflammation in neutrophil-driven autoimmune disease (16), while persistent neutrophil influx and NET formation exacerbate tissue damage during chronic inflammation (17). Strategies that direct neutrophils toward immunologically quiet, apoptotic resolution rather than inflammatory death therefore hold particular therapeutic relevance for resolving systemic and tissue inflammation.

Such precision is not achievable with current approaches. Although broad immune suppression through systemic corticosteroid treatment can alleviate neutrophil-mediated inflammatory pathology, this approach carries the significant cost of dampening anti-tumor immune responses (18). Thus, more precise strategies that selectively restrain pathogenic neutrophil activation without compromising anti-tumor immune responses are needed. A recent study was among the first to directly implicate changes in bioactive lipid mediators in the pathogenesis of neutrophil-driven severe ICB-irAEs (19). Longitudinal lipidomic profiling across cohorts of ICB-treated patients with solid tumors revealed that circulating lysophosphatidylcholines palmitoyl-lysophosphatidylcholine (LPC 16:0) and linoleoyl-lysophosphatidylcholine (LPC 18:2) were selectively reduced in individuals who developed colitis, pneumonitis, and other severe grade III/IV ICB-irAEs. Notably, reduced circulating LPC 18:2 correlated inversely with the increased peripheral neutrophilia observed in the severe ICB-irAE patients. LPC 18:2 supplementation *in vivo* reduced blood neutrophilia and attenuated tissue inflammation in murine models of ICB-induced colitis and dextran sulfate sodium (DSS)-induced colitis. Treated animals exhibited reduced disease severity, improved colon pathology, diminished weight loss, and preservation of colon length, indicating protection from inflammatory tissue injury. Importantly, LPC 18:2 supplementation did not impair anti-tumor responses during checkpoint blockade therapy, supporting a role for LPC 18:2 as an endogenous regulator of neutrophil-driven inflammation and immune homeostasis. Consistent with this association, supplementation with exogenous LPC 18:2, but not palmitoyl-, stearoyl-, or oleoyl-LPC (LPC 16:0, 18:0, or 18:1), attenuated blood and colonic neutrophilia and suppressed inflammatory pathology in murine models of ICB-irAEs and colitis.

LPCs constitute a class of circulating bioactive lipid mediators whose immunological functions are strongly influenced by acyl chain composition. Dysregulation of circulating LPC levels are reported across a wide range of inflammatory and infectious conditions (20–26). *In vitro* studies demonstrate that LPCs directly reprogram innate immune cells, including neutrophils, macrophages and endothelial cells, in an acyl chain–dependent manner (26, 27). Unsaturated LPCs are more potent inducers of neutrophil oxidative burst, while saturated species act more strongly on calcium signaling and membrane permeability (27).

Collectively, *in vivo* data demonstrating LPC 18:2-mediated protection against both neutrophilic colitis and neutrophilic ICB-irAEs support a protective role for LPC 18:2 in resolving neutrophil-driven pathologies across multiple inflammatory contexts. While both LPC 16:0 and LPC 18:2 were associated with reduced risk of severe ICB-irAEs, only LPC 18:2 inversely correlated with peripheral blood neutrophilia in patients receiving ICB therapy and only LPC 18:2 conferred protection in murine colitis models (19). We reasoned that this divergence may reflect acyl chain–dependent regulation of neutrophil fate. To test this hypothesis, here we perform functional *in vitro* assessments of human neutrophils treated with physiological concentrations of LPC 16:0 or LPC 18:2. These studies provide mechanistic insight into the functional, cell-intrinsic role of LPCs on primary neutrophil function *in vitro*, and support the potential for a targeted LPC 18:2–based strategy to restrain neutrophil-driven inflammation without compromising anti-tumor immunity, while highlighting the broader relevance of LPC 18:2 as a potential mediator of neutrophil-driven inflammation in disease.

## 2 Methods

### 2.1 Lysophosphatidylcholine preparation

1-Hexadecanoyl-sn-glycero-3-phosphocholine (palmitoyl-lysophosphatidylcholine, LPC 16:0) (Avanti Research, Cat# 855675C) and 1-Linoleoyl-2-hydroxy-sn-glycero-3-phosphocholine (linoleoyl-lysophosphatidylcholine, LPC 18:2) (Echelon Biosciences, Cat# L-1520) were used in all experiments. 1-Linoleoyl-2-hydroxy-sn-glycero-3-phosphate (linoleoyl-lysophosphatidic acid, LPA 18:2) (Avanti Research, Cat# 857138C) was used in experiments involving LPA 18:2. Lyophilized LPC or LPA powders were dissolved in chloroform to generate stock solutions at 25 mg/mL, which were stored at -20 °C in glass vials under inert argon gas until use. For all experiments, LPC or LPA solutions in chloroform were vacuum-evaporated in glass vials, and the resulting lipid films were reconstituted in prewarmed 37 °C RPMI 1640 Medium (ATCC modification) (Thermo Fisher Scientific, Cat# A1049101). LPC- or LPA-containing media were incubated at 37 °C for at least 30 minutes prior to use. All stocks were tested for endotoxin prior to use using the Pierce™ Chromogenic Endotoxin Quant Kit (Thermo Fisher Scientific, Cat# A39552).

### 2.2 Isolation of human neutrophils

The Miltenyi Whole Blood Neutrophil Isolation Kit (Miltenyi Biotec, Cat# 130-104-434) was used to negatively isolate human neutrophils from whole blood of healthy human donors according to the manufacturer’s instructions. This kit yields ≥97% neutrophil purity within the live leukocyte population, as reported by the manufacturer. Briefly, whole blood was incubated with reconstituted antibody-labeled beads at room temperature for 5 minutes then placed in a magnetic separator for 30 minutes at room temperature. Following incubation, the supernatant containing neutrophils was collected, and red blood cell lysis was performed for 5 minutes at room temperature. Neutrophils were subsequently resuspended in RPMI 1640 Medium (ATCC modification) supplemented with 5% heat-inactivated FBS and maintained at 37 °C until use.

### 2.3 Intracellular reactive oxygen species assay

0.4 × 10^6^ cells were seeded per well in a V-bottom 96-well tissue culture-treated plate. Cells were washed twice with FBS-free RPMI 1640 Medium (ATCC modification), then incubated with 100 µL of 10 µM dihydrorhodamine 123 (DHR, Thermo Fisher Scientific, Cat# D632) for 20 minutes at 37 °C to assess intracellular reactive oxygen species (ROS) production. After incubation, 100 µL of the respective test condition compounds (prepared at 2× concentration) were added directly to each well, resulting in a final volume of 200 µL. Cells were incubated under these conditions for 45 minutes or 2 hours at 37 °C. After treatment, plates were placed on ice for 5 minutes to halt cellular processes. Cells were then centrifuged at 300 × g for 5 minutes, and supernatants were discarded. In order to stain for apoptotic neutrophils, samples were washed twice with the Annexin V binding buffer (BioLegend) and stained using the Pacific Blue labeled Annexin V (BioLegend) as per instructions provided by the manufacturer (15 minutes at room temperature in the dark). Samples were finally resuspended in 200 µL of FACS buffer (1×PBS with 2% FBS + 0.05% Sodium Azide) containing propidium iodide (viability (PI, 1:200 dilution). Samples were immediately analyzed on an BD LSRFortessa™ X-20 Cell Analyzer (BD Biosciences) using the BD FACSDiva™ Software. The flow cytometry gating strategy used for analysis is shown in **Supplementary Figure 1**.

### 2.4 Caspase 3/7 activity assay

0.4 × 10^6^ cells were seeded per well in a V-bottom 96-well tissue culture-treated plate. Cells were washed twice with FBS-free RPMI 1640 Medium (ATCC modification). Following washes, cells were treated with 200 µL of the respective experimental condition compounds for the designated time periods. At the end of incubation, plates were placed on ice for 5 minutes to halt cellular activity. Cells were subsequently centrifuged at 300 × g for 5 minutes. Cell pellets were then resuspended in 100 µL of a 1:1000 dilution of caspase-3/7 detection reagent (as per the manufacturer’s instructions (Thermo Fisher Scientific, Cat# C10740) prepared in a pre-warmed (37°C) FACS buffer. Cells were incubated at 37°C for 25 minutes. Following incubation, 100 µL of SYTOX solution (1:500 dilution in FACS buffer) was added to each well and incubation was continued for an additional 5 minutes. Samples were analyzed using a BD^®^ LSR II Flow Cytometer (BD Biosciences) using the BD FACSDiva™ Software. The flow cytometry gating strategy used for analysis is shown in **Supplementary Figure 1**.

### 2.5 Neutrophil elastase activity assay

Neutrophil elastase activity was quantified using the Neutrophil Elastase Activity Assay Kit (Cayman Chemical Cat# 600610) according to the manufacturer’s instructions. Briefly, human neutrophils were seeded at 0.4 × 10^6^ cells per well in V-bottom 96-well plate in FBS-free RPMI 1640 Medium (ATCC modification). Cells were then treated with 200 µl of appropriate conditioned media and allowed to incubate at 37 °C for 45 minutes. Following treatment, culture plates were centrifuged at 300 × g for 5 minutes at room temperature, and cell-free supernatants were collected for analysis. Supernatants were transferred to a fresh black-bottom 96-well plate for analysis. Neutrophil elastase activity was then measured using the fluorogenic substrate [(Z-Ala-Ala-Ala-Ala)_2_Rh110] provided in the kit. Cleavage of the substrate by neutrophil elastase releases rhodamine 110 (R110), resulting in a fluorescent signal that was quantified using a fluorescence plate reader (excitation: 485 nm; emission: 525 nm). Fluorescence intensity was recorded as relative fluorescence units (RFU). Background fluorescence from cell-free control wells was subtracted where appropriate. Data are presented as RFU values for each treatment condition.

### 2.6 Citrullinated histone H3 detection by flow cytometry

Neutrophils were seeded at 0.4 × 10^6^ cells per well in V-bottom 96-well plates and subjected to the specified treatment conditions (200 µl per well) in FBS-free RPMI 1640 Medium (ATCC modification). Following treatment, cells were gently fixed with 0.4% paraformaldehyde (PFA) for 10 minutes at room temperature (20 µl of 4% PFA in 200 µl media). Cells were then spun at 300 × g for 3.5 minutes at room temperature and the supernatant was discarded. A second fixation was then performed using 100 µl/well of 4% PFA for 8 minutes at room temperature. To restrict detection to extracellular CitH3, cells were not permeabilized. Cells were then washed twice with PBS, blocked using the Human TruStain FcX (an Fc receptor blocking solution, BioLegend, Cat# 422301) as per manufacturer’s instructions and then stained overnight at 4 °C using a 1:300 dilution of the rabbit anti-citrullinated Histone H3 (R2, R8, R17) primary antibody (Abcam, Cat# ab5103). The antibody recognizes histone H3 citrullinated at arginine residues 2, 8, and 17 and has been widely used for detection of histone citrullination associated with NET formation and chromatin decondensation. Following incubation, cells were washed twice with FACS buffer and then stained with an Alexa Fluor™ 488-tagged secondary antibody (Thermo Fisher Scientific, Cat# A-11008) for 45 minutes at room temperature, in the dark. Following two washes, cells were analyzed by flow cytometry using the BD LSRFortessa™ X-20 Cell Analyzer (BD Biosciences) and data were processed using FlowJo™ Software. CitH3 expression was quantified as mean fluorescence intensity (MFI) of CitH3-positive cells. The flow cytometry gating strategy used for analysis is shown in **Supplementary Figure 1**.

### 2.7 CitH3 detection by immunoblotting

Isolated neutrophils from 4 donors were mixed together and seeded in deep-well 96-well plates (Axygen™, Cat# 14-222-224) at a density of 2 × 10^6^ cells per well in 1 mL of FBS-free RPMI 1640 Medium (ATCC modification), with or without test compounds (LPC 16:0 or LPC 18:2 at 10 μM), and incubated at 37 °C for 10 minutes. After treatment, cells and supernatant were transferred to microfuge tubes and spun down at 300 × g for 5 minutes at room temperature. Supernatant was removed, and cell pellet was directly lysed in 30 μL room temperature 1X NuPAGE LDS Sample Buffer (4X LDS Sample Buffer (Thermo Scientific, Cat# NP0007), 10X Reducing Agent (Thermo Scientific, Cat# NP0009), rest ddH_2_O). Sample lysate was combined in duplicate for a total of 4 million treated cells per condition then heated at 70 °C for 10 minutes prior to freezing at -20 °C until use. Before loading, samples were sheared by passing them through the needle of a 29-gauge needle approximately 10 times. Sample preparation, gel electrophoresis, transfer and blocking were performed as previously described (28). Blots were incubated with anti-histone H3 (citrulline R2 + R8 + R17) antibody (1:1000, Abcam, Cat# ab5103) at 4 °C overnight or GAPDH antibody (1:2000, Cell Signaling Technology, Cat# 2118) at 22 °C for 1 hour. Blots were washed with Tris-buffered Saline-0.1% with Tween 20 and incubated with horseradish peroxidase-conjugated anti-rabbit IgG secondary antibody (1:5000, Sigma-Aldrich, Cat# A0545) at 22 °C for 1 hour. Signal was detected using the Enhanced Chemiluminescence (ECL) Reagent (Bio-Rad, Cat# 1705061) and imaged on the Bio-Rad ChemiDoc.

### 2.8 Lactate dehydrogenase assay

Cytotoxicity was measured using the CyQUANT™ LDH Cytotoxicity Assay Kit (Thermo Fisher Scientific, Cat# C20300) according to the manufacturer’s instructions. Cells were plated in 96-well V-bottom plates and incubated at 37 °C for 1.5 hours with treatment compounds. After treatment, 50 µL of culture supernatant from each well was transferred to a clear flat-bottom 96-well plate. Cells were lysed with 10× Lysis Buffer. Reaction Mixture 50 µL was added to each well and incubated at room temperature for 30 minutes, protected from light. The reaction was stopped by adding 50 µL of Stop Solution, and absorbance was measured at 490 nm with 680 nm as reference.

### 2.9 Extracellular high mobility group box 1 release assay

Extracellular HMGB1 release was quantified using the Lumit™ HMGB1 (Human/Mouse) Immunoassay (Promega, Cat# W6110) according to the manufacturer’s instructions. Human neutrophils were washed and seeded at 0.4 × 10^6^ cells/well in a V-bottom plates and treated under the specified experimental conditions in FBS-free RPMI 1640 Medium (ATCC modification). Following treatment, cell-free supernatants were collected and 80 µL of each sample was transferred to a white 96-well assay plate. HMGB1 standards were prepared by serial dilution of recombinant HMGB1 positive control in the same culture medium used for experimental samples. Samples and standards were incubated with 20 µL of the antibody mixture containing SmBiT- and LgBiT-conjugated anti-HMGB1 antibodies and incubated for 90 minutes at room temperature. Subsequently, 25 µL of Lumit™ Detection Reagent B was added to each well and plates were incubated for 5 minutes at room temperature. Luminescence was measured using a microplate reader and recorded as relative luminescence units (RLU). Background signal from medium-only control wells was subtracted from all measurements prior to analysis.

### 2.10 Water-soluble tetrazolium salt-1 assay

Cells were seeded in 96-well plates at a density of 0.4 × 10^6^ cells in 100 µL of FBS-free RPMI 1640 Medium (ATCC modification), with or without test compounds, and incubated at 37 °C for 1 hour. Following incubation, 10 µL of WST-1 reagent (Cayman Chemical Company, Cat# 10008883) was added to each well, and plates were gently mixed on an orbital shaker for 1 minute. Cells were then incubated for 4 hours at 37 °C. Prior to measurement, plates were mixed again for 1 minute. Absorbance was measured at 450 nm using a microplate reader.

### 2.11 Myeloperoxidase activity assay

Myeloperoxidase (MPO) activity was measured using the Neutrophil Myeloperoxidase Activity Assay Kit (Cayman Chemical Company, Cat# 600620) according to the manufacturer’s instructions. Briefly, cells were plated in a 96-well V-bottom plate and treated with test compounds at 37 °C for 45 minutes in 200 μL media/well. After treatment, the plate was centrifuged at 1200 rpm for 10 minutes at room temperature. 25 μL of culture supernatant from each well was transferred to the assay plate to probe for MPO activity. 25 μL of assay buffer was then added to each well, followed by 50 μL of the TMB substrate solution. Plate absorbance was read at 650 nm at one and five minutes after the addition of substrate. Change in absorbance per minute and myeloperoxidase activity in μmole/min/mL were calculated according to the manufacturer’s instructions.

### 2.12 Bulk RNA sequencing

Cells were seeded in deep-well 96-well plates (Axygen™, Cat# 14-222-224) at a density of 2 × 10^6^ cells in 1 mL of FBS-free RPMI 1640 Medium (ATCC modification), with or without test compounds (LPC 16:0 or LPC 18:2 at 10 μM), and incubated at 37 °C for 45 minutes or 2 hours. Total RNA was extracted from 2 million cells using the Direct-zol RNA Miniprep Kit (Zymo Research, Cat# R2050). Libraries were prepared using the NEB Ultra II Directional RNA Library Prep Kit for Illumina with the NEBNext Poly(A) mRNA Magnetic Isolation Module. 20 ng total RNA was used for each sample and libraries underwent 16 cycles of PCR amplification. Agilent Universal Human Reference RNA was prepared as a process control. Libraries were sequenced on the Illumina Novaseq 6000 using a 50×50 run configuration at a targeted depth of 25M (50M paired-end) reads.

### 2.13 RNA-seq analysis pipeline

The paired-end reads that passed Illumina filters were filtered for reads aligning to tRNA, rRNA, adapter sequences, and spike-in controls. The reads were then aligned to GRCh38 reference genome and Gencode v27 annotations using STAR (v 2.6.1) (29). DUST scores were calculated with PRINSEQ Lite (v0.20.3) (30) and low-complexity reads (DUST >4) were removed from the BAM files. The alignment results were parsed via the SAMtools (31) to generate SAM files. Read counts to each genomic feature were obtained with featureCount (v 1.6.5) (32). After removing absent features (zero counts in all samples), the raw counts were then imported to R/Bioconductor package DESeq2 (v 1.24.0) (33) to identify differentially expressed genes among samples. P-values for differential expression were calculated using the Wald test for differences between the base means of two conditions. These P-values were then adjusted for multiple test correction using the Benjamini Hochberg algorithm (34). Gene set enrichment analysis was run using the ‘GseaPreranked’ method with ‘classic’ scoring scheme. MSigDB Gene sets were downloaded from the MSigDB website. Rank files for each DE comparison of interest were generated by assigning a rank of negative log10(pValue) to genes with log2FoldChange greater than zero and a rank of positive log10(pValue) to genes with log2FoldChange less than zero. Significantly differentially expressed transcripts not known to be expressed in mature human neutrophils (*HBA2*, *HBB*, *NKG7*, etc.) were considered contaminants from other blood cell populations and were excluded from DEG analysis.

### 2.14 Lipid-raft disruption

Cells were seeded at 0.4 × 10^6^ cells per well in a 96-well V-bottom plate, centrifuged and then resuspended with Methyl-β-cyclodextrin (MβCD, Sigma-Aldrich, Cat# C4555) at final concentrations of 500 µM or 1 mM to disrupt cholesterol-rich lipid raft microdomains. A total of 100 µL of the MβCD solution was added per well, and cells were incubated at 37 °C for 35 minutes prior to treatment with test compounds.

### 2.15 Cytochrome *c* release assay

To test for cytochrome *c* release, a widely used readout of mitochondrial integrity and apoptotic cell death, post-treatment with test compounds, cells were fixed with 100 µl/well 4% PFA for 10 minutes at room temperature. Following fixation, cells were washed with 1×PBS, and then permeabilized with 0.1% PBS-TritonX-100 for 15 minutes at room temperature. After incubation, cells were washed with 1×PBS and blocked with 10% Goat serum in 1×PBS for 30 minutes at room temperature. Next, the cells were stained with Cytochrome *C* Monoclonal Antibody FITC (eBioscience, Cat# 11-6601-82) at a 1:100 concentration in the blocking buffer and left to incubate for 8 hours at 4 °C. Cells were then washed and resuspended in 1×PBS and analyzed using the BD LSR II Fortessa™ Cell Analyzer (BD Biosciences). The flow cytometry gating strategy used for analysis is shown in **Supplementary Figure 1**.

### 2.16 Mitochondrial potential assay (MiTorracker Red assay)

The MitoTracker Red assay is used to assess mitochondrial membrane potential, a key indicator of mitochondrial health. The dye accumulates in active mitochondria in a potential-dependent manner, so loss of fluorescence reflects mitochondrial depolarization and subsequent dysfunction, which occurs as a result of intrinsic apoptosis. Briefly, after incubation with test compounds, neutrophils were spun down, supernatant removed, and the cells were resuspended with 100 µL of RPMI 1640 Medium (ATCC modification) containing a final concentration of 50 nM MitoTracker Red CMXRos (Thermo Fisher Scientific, Cat# M7512). Cells were then incubated at 37 °C for 35 minutes. Finally, cells were resuspended in FACS buffer containing 1:8000 DAPI and then analyzed on the BD LSR II Fortessa™ Cell Analyzer (BD Biosciences). The flow cytometry gating strategy used for analysis is shown in **Supplementary Figure 1**.

### 2.17 Mitochondrial ROS and total intracellular ROS (MitoSOX Red and DHR/ROS assay)

Total ROS production and mitochondrial reactive oxygen species (mtROS) production were assessed using MitoSOX Red mitochondrial superoxide indicator (Thermo Fisher Scientific, Cat# M36008) and dihydrorhodamine 123 (DHR, Thermo Fisher Scientific, Cat# D632). Neutrophils were seeded at a density of 0.4 × 10^6^ cells per well. Cells were treated with DHR-containing FBS-free RPMI 1640 Medium (ATCC modification) (+/- 100 µM MitoTEMPO, Sigma-Aldrich, Cat# SML0737) at 100 µl per well and incubated at 37 °C for 15 minutes. At the end of incubation, cells were treated under the indicated experimental conditions (+/- 100 µl of MitoTEMPO) in FBS-free RPMI 1640 Medium (ATCC modification) (at 2× concentration, 100 µl of media per well) and incubated at 37 °C for 45 minutes. Following treatment, cells were incubated with MitoSOX Red working solution in Ca2+Mg2+ HBSS buffer according to the manufacturer’s instructions for 30 minutes at 37 °C, protected from light. Cells were subsequently washed with Ca+ Mg+ HBSS buffer. Fluorescence was measured by flow cytometry using the BD LSRFortessa™ X-20 Cell Analyzer (BD Biosciences), and data were analyzed using FlowJo™ Software. mtROS was quantified as the percentage of MitoSOX-positive cells while total ROS was quantified as mean fluorescence intensity (MFI) of DHR-positive cells. The flow cytometry gating strategy used for analysis is shown in **Supplementary Figure 1**.

### 2.18 Statistical analysis

Statistical analyses for bulk RNA-sequencing data were performed in R. Statistical analyses for *in vitro* experiments were conducted using GraphPad Prism. Ordinary one-way or two-way analysis of variance (ANOVA) was used for comparisons involving more than two groups or multiple factors, respectively, with appropriate *post hoc* multiple-comparison testing (Dunnett’s test or Tukey’s test) where indicated. All experiments were independently replicated a minimum of three times. Data are presented as mean ± SEM.

## 3 Results

### 3.1 LPC 16:0 induces lytic cell death distinct from LPC 18:2

Plasma lipidomics profiling of ICB-treated patients previously revealed that individuals who developed severe ICB-irAEs exhibited significantly lower circulating concentrations of LPC 16:0 and LPC 18:2 compared with patients who experienced mild ICB-irAEs or did not develop ICB-irAEs, suggesting a protective inverse association between LPC abundance and systemic inflammation (19). LPC 18:2 correlated inversely with peripheral blood neutrophil counts in ICB-treated patient cohorts, whereas LPC 16:0 showed no such correlation, and only supplementation of LPC 18:2 abrogated ICB-irAE development in murine models by limiting blood and tissue neutrophilia. These observations motivated investigation into the distinct effects of saturated and unsaturated LPC species on neutrophil fate *in vitro*.

LPCs are among the most abundant lysophospholipids in plasma (35), with total LPC concentrations typically in the range of ~100–300 µM. LPC 16:0 and LPC 18:2 are two of the most abundant LPCs in this pool, with LPC 16:0 levels at approximately 20–40 µM and LPC 18:2 at 10–25 µM in humans and mice (19, 27). In a recent study (19), circulating LPC 18:2 and LPC 16:0 were directly quantified in patient cohorts to have baseline concentrations of ~30 µM and, after the onset of severe ICB-irAEs, diminished to ~10 µM. To assess the effects of LPC acyl chain composition on neutrophils, primary neutrophils were isolated from the peripheral blood of healthy human donors and exposed to these physiologically relevant concentrations (10, 30 µM) of LPCs 16:0 and 18:2.

**Figure 1:**
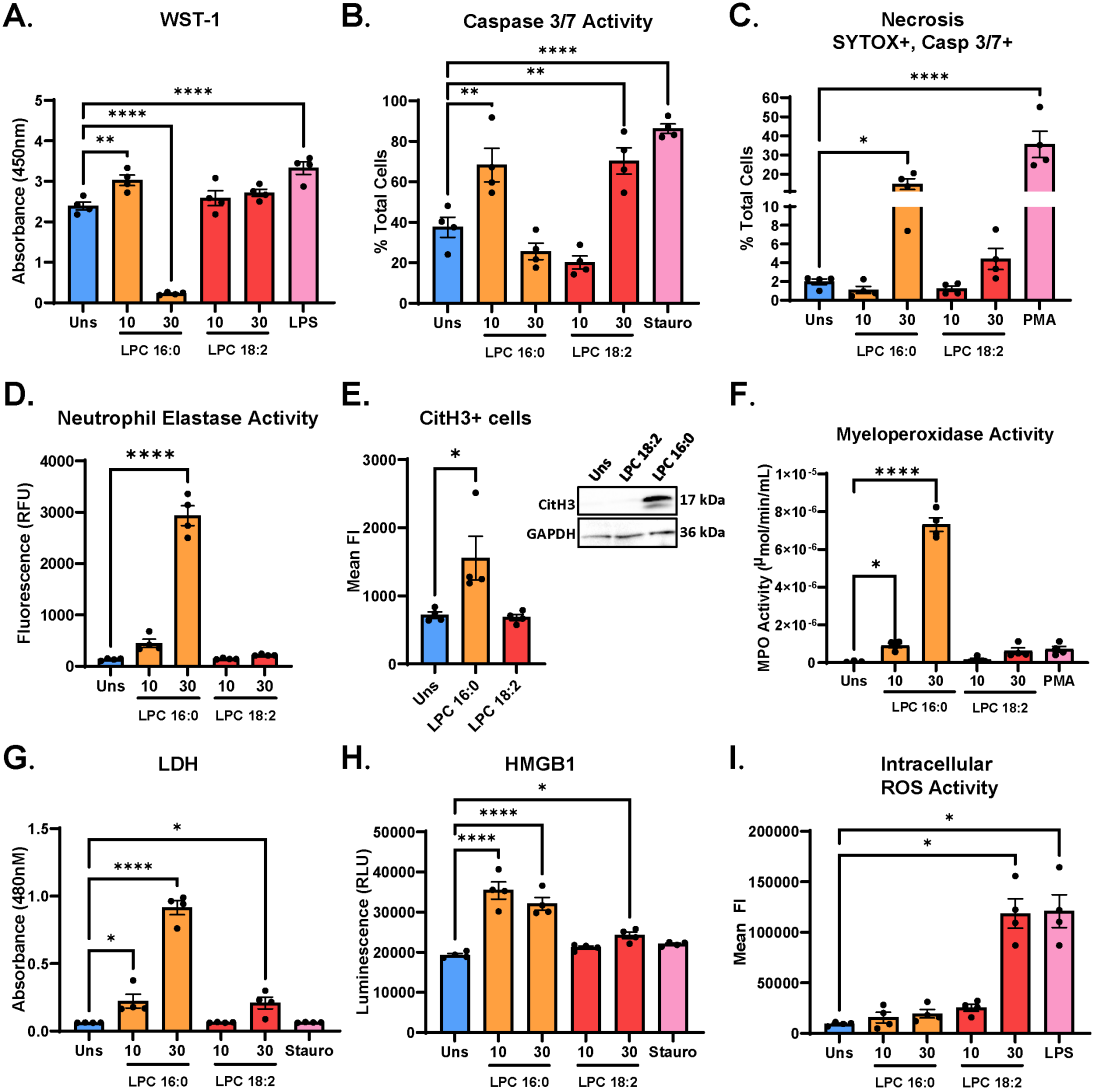
LPC 16:0 and LPC 18:2 differentially modulate human neutrophil phenotype. **(A)** Human neutrophil cellular metabolic activity measured by water-soluble tetrazolium salt-1 (WST-1) assay after treatment with medium alone (Uns), LPC 16:0 (10, 30 μM), LPC 18:2 (10, 30 μM), or Lipopolysaccharide (LPS, 1 μg/mL) for 1 hour. **(B)** Human neutrophil apoptosis (cleaved caspase-3/7) after treatment with medium alone (Uns), LPC 16:0 (10, 30 μM), LPC 18:2 (10, 30 μM), or Staurosporine (Stauro, 3 μM) for 2 hours. **(C)** Human neutrophil necrosis (SYTOX+ caspase-3/7 dual stain) after treatment with medium alone (Uns), LPC 16:0 (10, 30 μM), LPC 18:2 (10, 30 μM), or Phorbol 12-myristate 13-acetate (PMA, 5 nM) for 2 hours. **(D)** Neutrophil elastase activity in human neutrophils treated with medium alone (Uns), LPC 16:0 (10, 30 μM) or LPC 18:2 (10, 30 μM) for 45 minutes measured by fluorogenic substrate cleavage in relative fluorescence units (RFU). **(E)** Citrullinated histone H3 (CitH3) in human neutrophils treated with medium alone (Uns), LPC 16:0 (10 μM) or LPC 18:2 (10 μM), quantified as extracellular CitH3 by flow cytometry (mean fluorescence intensity, left) after treatment for 20 minutes and total CitH3 by immunoblot with GAPDH as a loading control after treatment for 10 minutes (right). **(F)** Myeloperoxidase activity in human neutrophils treated with medium alone (Uns), LPC 16:0 (10, 30 μM), LPC 18:2 (10, 30 μM), or PMA (100 nM) for 45 minutes. **(G)** Lactate Dehydrogenase (LDH) release by human neutrophils after treatment with medium alone (Uns), LPC 16:0 (10, 30 μM), LPC 18:2 (10, 30 μM), or Staurosporine (3 μM) for 1.5 hours. **(H)** High-Mobility Group Box 1 (HMGB1) release by human neutrophils after treatment with medium alone (Uns), LPC 16:0 (10, 30 μM), LPC 18:2 (10, 30 μM), or Staurosporine (3 μM) for 2 hours. **(I)** Intracellular ROS induction in human neutrophils treated with medium alone (Uns), LPC 16:0 (10, 30 μM), LPC 18:2 (10, 30 μM), or LPS (1 μg/mL) for 2 hours, measured by dihydrorhodamine 123 (DHR) mean fluorescence intensity. Statistics: Ordinary one-way ANOVA with Dunnett’s test **(A–I)**; in all panels, bars = mean, error = SEM. (*p ≤ 0.05, **p ≤ 0.01, ***p ≤ 0.001, ****p ≤ 0.0001).

Cellular metabolic activity was quantified using the WST-1 assay, which measures extracellular reduction of tetrazolium salts by plasma membrane NADPH oxidoreductases and intact mitochondrial redox systems. Treatment with LPC 16:0 at 10 µM resulted in a ~25% increase in WST-1 signal (**Figure 1A**). Given that WST-1 reduction depends on mitochondrial and cytosolic dehydrogenase activity and that neutrophils are terminally differentiated, non-dividing cells, this increase likely reflects elevated metabolic activity rather than proliferation (36). In contrast, treatment with LPC 16:0 at 30 µM led to a marked ~90% reduction in WST-1 signal, consistent with rapid loss of redox capacity and disruption of membrane integrity (37). This pronounced decrease suggests that higher doses of LPC 16:0 induce early metabolic collapse consistent with lytic cell death. By comparison, LPC 18:2 treatment produced no detectable change in WST-1 signal at 10 µM and a modest, non-significant increase at 30 µM, indicating preservation of metabolic activity across physiological concentrations. Treatment with either LPC species significantly increased caspase-3/7 activity, suggesting engagement of apoptotic pathways (**Figure 1B**; gating strategy in **Supplementary Figure 1**). Specifically, while 10 µM LPC 16:0 induced apoptosis, treatment with 30 µM LPC 16:0 demonstrated significant loss of viability consistent with necrotic cell death as measured by increased SYTOX/caspase-3/7^+^ staining (**Figure 1C**; gating strategy shown in **Supplementary Figure 1**). Caspase-3/7 activity was also significantly elevated in neutrophils treated with 30 µM LPC 18:2, although cell viability was not significantly changed, indicating differential regulation of cell death pathways by the two LPC species (**Figure 1B**).

With a marked increase in SYTOX positivity following 30 µM LPC 16:0 treatment, we next assessed whether LPC treatment induced neutrophil extracellular trap (NET)-related pathways. As neutrophil elastase (NE) is a key mediator of chromatin decondensation during NETosis and its extracellular release is commonly associated with inflammatory neutrophil death, we quantified NE activity after LPC treatment. LPC 16:0 at 10 μM produced a modest ~3–4-fold increase in NE activity that did not reach statistical significance, while treatment with 30 μM LPC 16:0 induced a robust ~20–25-fold increase relative to unstimulated controls (**Figure 1D**). LPC 18:2 (10 and 30 μM) did not significantly alter NE activity relative to unstimulated controls (**Figure 1D**).

To assess for extracellular chromatin modification associated with NET formation, neutrophils treated with LPC species at 10 µM were stained for extracellular citrullinated histone H3 (CitH3) under non-permeabilizing conditions as well as profiled for total CitH3 through immunoblotting. LPC 16:0 treatment significantly increased surface-associated CitH3 (approx. 2-fold increase) relative to unstimulated neutrophils, with a corresponding increase in total CitH3 by immunoblot (**Figure 1E**; gating strategy in **Supplementary Figure 1**). This indicates enhanced histone citrullination and NET formation. In contrast, LPC 18:2 did not significantly alter extracellular or total CitH3 relative to unstimulated controls.

Activated neutrophils release azurophilic granules through degranulation, a process that contributes to inflammatory tissue damage and which often accompanies NET formation. We therefore measured if LPC treatment drives extracellular release of myeloperoxidase (MPO), an azurophilic granule enzyme and a hallmark of neutrophil activation. Consistent with the release of granule contents, LPC 16:0 significantly increased extracellular MPO activity at both 10 µM and 30 µM, whereas neither concentration of LPC 18:2 produced a significant change (**Figure 1F**). These findings demonstrate that LPC 16:0 promotes substantial extracellular elastase release and neutrophil degranulation, consistent with activation of a lytic, pro-inflammatory cell death program with DAMP release.

To further explore LPC-mediated lytic cell death, we measured lactate dehydrogenase (LDH) and high-mobility group box 1 (HMGB1) release, indicators of membrane rupture and lytic cell death. At 1.5 hours post-treatment, LDH release increased nearly 10-fold in 30 µM LPC 16:0–treated neutrophils, indicating extensive membrane damage (**Figure 1G**), compared with less than a 2-fold increase following 30 µM LPC 18:2 treatment. At 2 hours post-treatment, HMGB1 release was elevated 1.8-fold and 1.6-fold following LPC 16:0 treatment at 10 µM and 30 µM, respectively, relative to unstimulated controls (**Figure 1H**). In contrast, 30 µM LPC 18:2 induced only mild (approx 1.2-fold) HMGB1 release. To assess oxidative stress responses underlying the observed differences in metabolic activity and cell death, we quantified intracellular ROS production by flow cytometry in live neutrophils using the oxidation-sensitive fluorescent probe dihydrorhodamine 123 (DHR), excluding late apoptotic Annexin V^+^ cells in order to focus specifically on viable populations (**Figure 1I**; gating strategy shown in **Supplementary Figure 1**). Consistent with prior reports (27), LPC 18:2 treatment induced a robust ~13-fold increase in intracellular ROS production, whereas LPC 16:0 elicited modest and statistically insignificant increase (**Figure 1I**).

Collectively, these findings demonstrate that LPC 16:0 promotes a rapid, inflammatory, and lytic neutrophil death program characterized by histone citrullination and chromatin release, granule enzyme release, membrane disruption, and metabolic collapse, consistent with NETosis-like cell death. In contrast, LPC 18:2 does not induce appreciable NET formation despite triggering neutrophil death, instead promoting robust ROS generation and caspase-3/7 activation with comparatively limited membrane rupture, consistent with a non-lytic apoptotic program.

### 3.2 Bulk RNA sequencing reveals pro-inflammatory transcriptional responses to LPC 16:0 treatment compared with LPC 18:2 treatment

**Figure 2:**
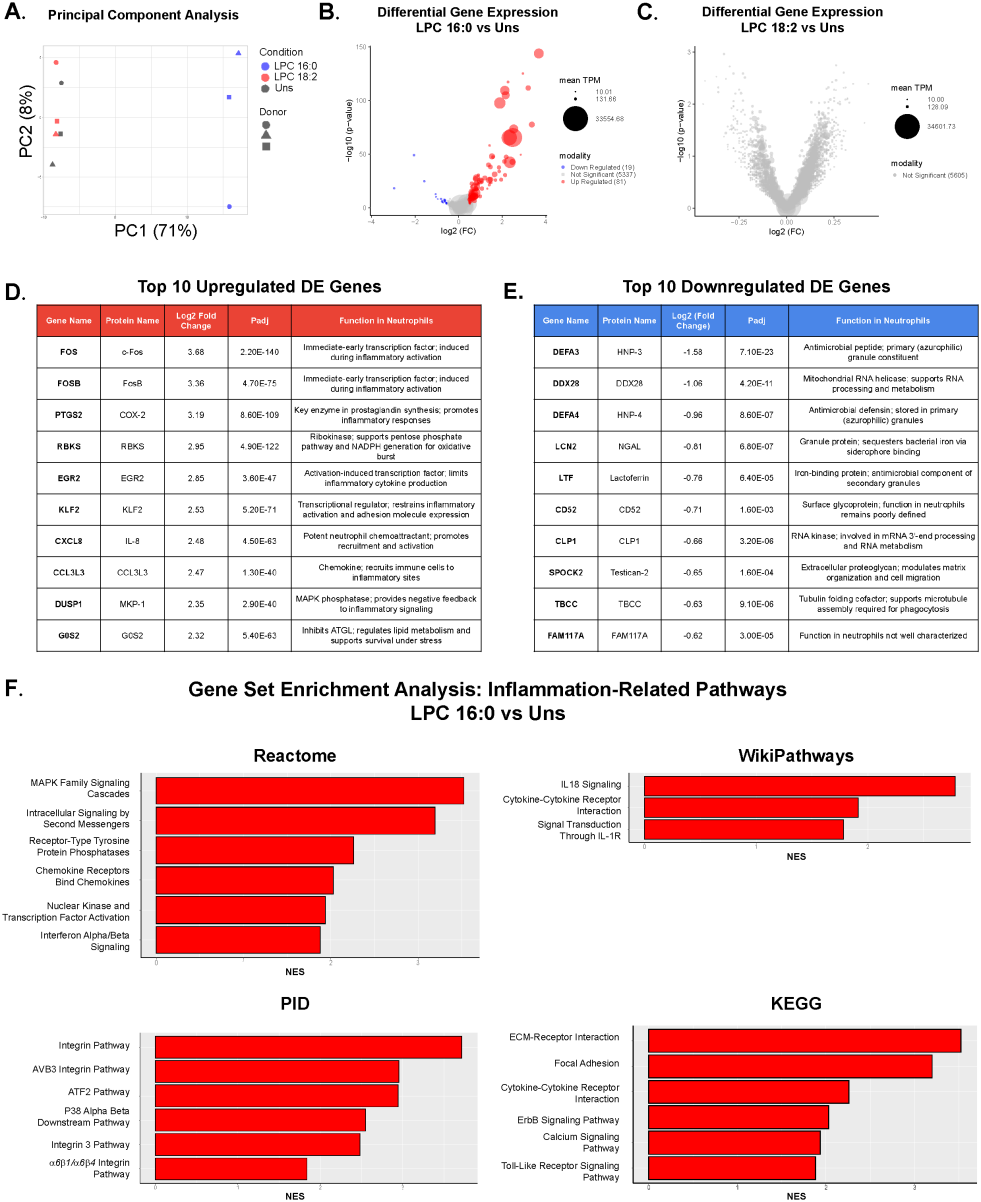
LPC 16:0 but not LPC 18:2 upregulates inflammatory neutrophil gene expression. **(A)** Principal component analysis (PCA) of human neutrophils (n = 3) treated with medium alone (Uns), LPC 16:0 (10 μM), or LPC 18:2 (10 μM) for 45 minutes. **(B)** Volcano plot of differentially expressed protein-coding genes (DEGs) between LPC 16:0-treated neutrophils and unstimulated cells (DESeq2). DEG significance threshold: FDR ≤ 0.01, |log_2_FC| ≥ 0.5, mean TPM > 10. **(C)** Volcano plot of DEGs between LPC 18:2-treated neutrophils and unstimulated cells (DESeq2). DEG significance threshold: FDR ≤ 0.01, |log_2_FC| ≥ 0.5, mean TPM > 10. **(D)** Top 10 significantly upregulated neutrophil-related genes after LPC 16:0 treatment. **(E)** Top 10 significantly downregulated neutrophil-related genes after LPC 16:0 treatment. **(F)** Inflammation-related gene pathways upregulated after LPC 16:0 treatment identified by gene set enrichment analysis (Reactome, WikiPathways, PID, and KEGG).

To define the molecular basis underlying differential neutrophil responses to LPC 16:0 and LPC 18:2, we performed bulk RNA sequencing on primary human donor neutrophils (n = 3) following 45-minute exposure to each LPC species at 10 µM, a timepoint and concentration chosen to capture early transcriptional events preceding cell death. Principal component analysis (PCA) revealed clear segregation of LPC 16:0–treated neutrophils from the unstimulated controls, whereas LPC 18:2–treated cells clustered closely with control samples (**Figure 2A**). Differential gene expression analysis identified 81 upregulated and 19 downregulated protein-coding genes in the LPC 16:0–treated neutrophils relative to unstimulated controls (FDR ≤ 0.01, |log_2_FC| ≥ 0.5, mean TPM > 10) (**Figure 2B**). In contrast, no transcripts met significance thresholds following LPC 18:2 treatment at 45 minutes (**Figure 2C**). Among the most highly upregulated genes in response to LPC 16:0 were inflammatory mediators including *FOS*, *FOSB*, *PTGS2*, *CXCL8*, *CCL3L3*, and *DUSP1*, which are collectively involved in neutrophil activation, cytokine production and survival (**Figure 2D**). Prominent downregulated genes included *DEFA3*, *DEFA4*, *LCN2*, and *LTF*, which encode granule-associated proteins involved in neutrophil degranulation and effector responses (**Figure 2E**). At a 2-hour timepoint, LPC 18:2 induced a modest transcriptional program consisting of 16 upregulated and 9 downregulated protein-coding genes relative to unstimulated controls (FDR ≤ 0.01, |log_2_FC| ≥ 0.5, mean TPM > 10). Upregulated genes included interferon-induced IFIT-family genes (*IFIT1, IFIT2, IFIT3, IFIT5, IRF9*) and downregulated genes included *GZMB* and multiple ribosomal protein genes (**Supplementary Figure 2**). This distinct type-I Interferon program differs from the immediate-early inflammatory signature elicited by LPC 16:0 at 45 minutes and indicates that the two lipids drive different transcriptional programs with different kinetics.

Pathway enrichment analyses using WikiPathways, Reactome, PID, and KEGG databases confirmed significant enrichment of inflammatory and cellular stress–response pathways in LPC 16:0–treated neutrophils at the 45-minute treatment timepoint (**Figure 2F**). Enriched programs included TNF-related signaling, IL-1 family signaling, cytokine–cytokine receptor interactions, MAPK activation, and NF-κB–dependent transcriptional responses, collectively indicating a broad pro-inflammatory transcriptional signature, consistent with results of functional assays in **Figure 1**.

### 3.3 LPC 18:2 induces intrinsic apoptosis

**Figure 3:**
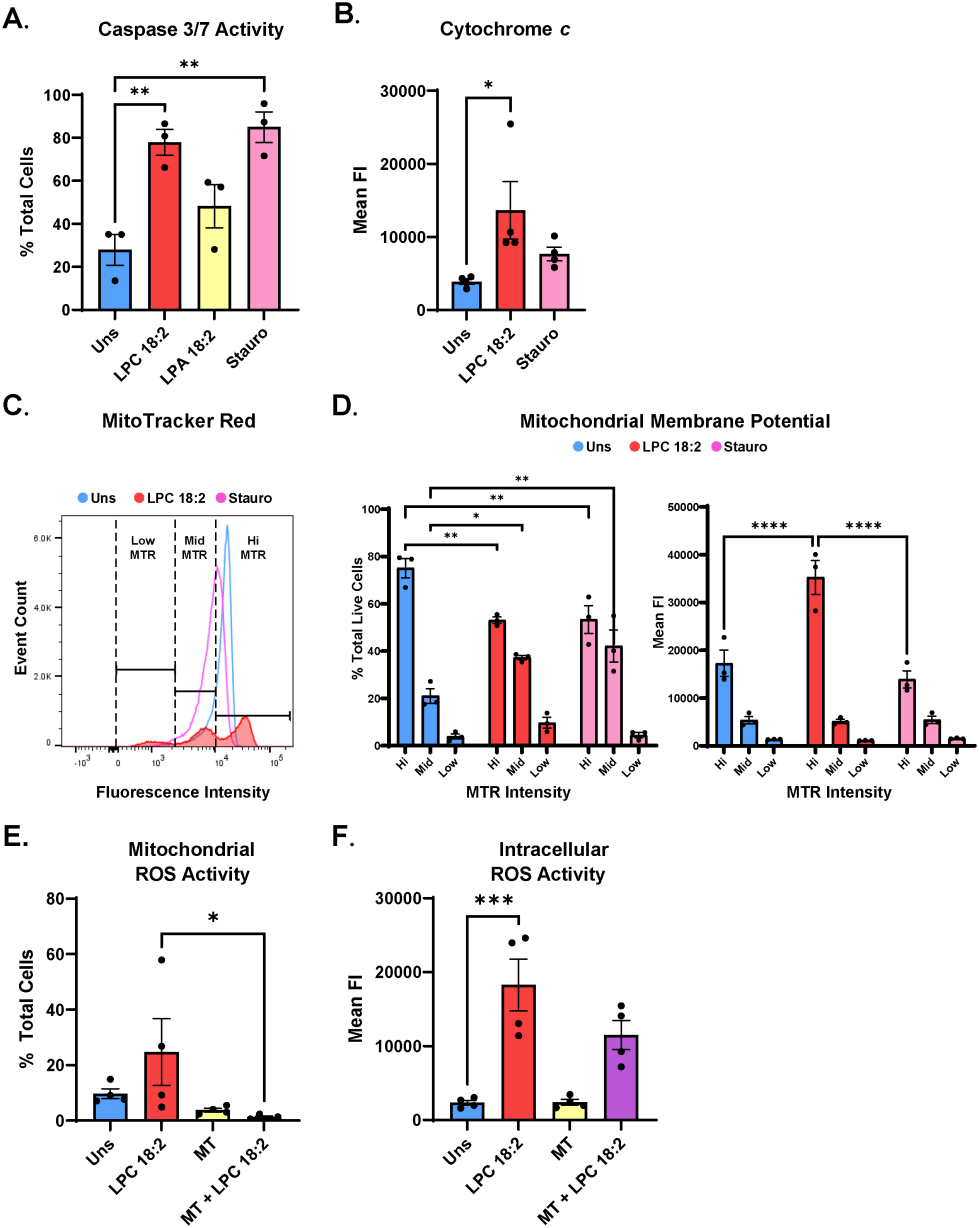
LPC 18:2 induces intrinsic apoptosis. **(A)** Human neutrophil apoptosis (cleaved caspase-3/7) after treatment with medium alone (Uns), LPC 18:2 (30 μM), LPA 18:2 (30 μM), or Staurosporine (Stauro, 3 μM) for 2 hours. **(B)** Cytochrome *c* release by human neutrophils after treatment with medium alone (Uns), LPC 18:2 (30 μM) or Staurosporine (Stauro, 3 μM) for 45 minutes. **(C)** MitoTracker Red fluorescence intensity of human neutrophils after treatment with medium alone (Uns), LPC 18:2 (30 μM) or Staurosporine (Stauro, 3 μM). Three populations were observed after LPC 18:2 treatment (Low MTR, Mid MTR, and Hi MTR). **(D)** Quantification of % total live cells and mean fluorescence intensity (MFI) of populations from 3C. **(E)** Mitochondrial ROS activity of human neutrophils after treatment with medium alone (Uns), LPC 18:2 (30 μM), MitoTEMPO (MT, 100 μM), or MitoTEMPO and LPC 18:2 for 45 minutes. mtROS was quantified as the percentage of MitoSOX-positive cells. **(F)** Intracellular ROS activity of human neutrophils after treatment with medium alone (Uns), LPC 18:2 (30 μM), MitoTEMPO (MT, 100 μM), or MitoTEMPO and LPC 18:2 for 45 minutes. Total ROS was quantified as MFI of DHR-positive cells. Statistics: Ordinary one-way ANOVA with Dunnett’s test (A, B, E, F), two-way ANOVA with Tukey’s test **(D)**; in all panels, bars = mean, error = SEM. (*p ≤ 0.05, **p ≤ 0.01, ***p ≤ 0.001, ****p ≤ 0.0001).

LPC 18:2 can serve as a substrate for the secreted enzyme autotaxin (ATX; lysophospholipase D), which catalyzes its conversion into linoleoyl-lysophosphatidic acid (LPA 18:2). LPA is a bioactive lipid mediator with established roles in inflammation and neutrophil activation (38). Although LPC 18:2 can be enzymatically converted to LPA 18:2, we observed that neutrophil apoptosis in our experimental system was driven specifically by LPC 18:2 and not by LPA 18:2, as treatment with LPA 18:2 did not increase caspase-3/7 activity (**Figure 3A**). These data indicate that pro-apoptotic effects observed are attributable to LPC 18:2 itself rather than its downstream conversion to LPA 18:2.

Two principal apoptotic pathways have been defined: the extrinsic pathway, initiated by death receptor signaling at the plasma membrane, and the intrinsic (i.e. mitochondrial) pathway, which is governed by mitochondrial integrity and release of cytochrome *c* into the cytosol. To determine whether LPC 18:2 induces intrinsic apoptosis in neutrophils, we assessed cytochrome *c* release and mitochondrial membrane potential changes. Treatment of primary human neutrophils with 30 µM LPC 18:2 resulted in a marked increase in cytosolic cytochrome *c* levels, consistent with mitochondrial outer membrane permeabilization (**Figure 3B**; gating strategy shown in **Supplementary Figure 1**). In parallel, mitochondrial membrane potential was assessed by flow cytometry using MitoTracker staining, with dead cells excluded by DAPI gating to restrict analysis to viable neutrophils (**Figure 3C, D**; gating strategy shown in **Supplementary Figure 1**). Staurosporine treatment induced a uniform loss of mitochondrial membrane potential, serving as a positive control for intrinsic apoptosis (**Figure 3C, D**, left). Following treatment with LPC 18:2, three distinct MitoTracker fluorescence populations emerged, designated High (Hi), Medium (Mid), and Low (Lo), representing graded levels of mitochondrial polarization (**Figure 3C**). Quantification of the DAPI^-^ population revealed that LPC 18:2 significantly reduced overall mitochondrial membrane potential, as evidenced by a decrease in the Hi population and a concomitant increase in the Mid population (**Figure 3D**). Notably, the mean fluorescence intensity (MFI) of MitoTracker within the remaining Hi population increased following LPC 18:2 treatment, a pattern not observed with Staurosporine (**Figures 3D, right**). This increase is consistent with elevated mitochondrial activity and ROS generation in the subset of live, non-apoptotic neutrophils, and is in agreement with the robust ROS induction observed following LPC 18:2 treatment (**Figure 1I**).

Given the evidence for mitochondrial dysfunction following LPC 18:2 treatment, we next assessed mitochondrial ROS production using MitoSOX Red, a mitochondria-targeted fluorogenic probe that selectively detects mitochondrial superoxide. Neutrophils were treated with LPC 18:2 (30 µM) for 45 minutes in the presence or absence of the mitochondrial ROS scavenger MitoTEMPO. LPC 18:2 treatment produced a modest increase in MitoSOX-positive cells, indicative of mitochondrial superoxide generation, although this increase did not reach statistical significance (**Figure 3E**; gating strategy shown in **Supplementary Figure 1**). Pretreatment with MitoTEMPO abolished the LPC 18:2-induced MitoSOX signal, confirming effective scavenging of mitochondrial ROS (p < 0.05). In contrast, MitoTEMPO did not significantly reduce the robust increase in total intracellular ROS measured by DHR fluorescence following LPC 18:2 treatment (**Figure 3F**; gating strategy in **Supplementary Figure 1**). Our findings show that LPC 18:2 does not induce significant mitochondrial ROS, which therefore does not contribute to the overall ROS response elicited by LPC 18:2. Further investigation is required to identify the dominant source of LPC 18:2-induced ROS activity.

Together with activation of caspase-3/7, executioner caspases downstream of cytochrome *c* signaling, these findings demonstrate that LPC 18:2 triggers a mitochondria-dependent intrinsic apoptotic program in neutrophils. Although LPC 18:2 induces substantial intracellular ROS accumulation (**Figures 1I**, **3F**), our data suggest that this ROS originates predominantly from non-mitochondrial sources. This program contrasts sharply with LPC 16:0, which drives early metabolic collapse, membrane rupture, and lytic cell death.

### 3.4 Lipid raft stability is integral for LPC 18:2 driven ROS activity and apoptosis

**Figure 4:**
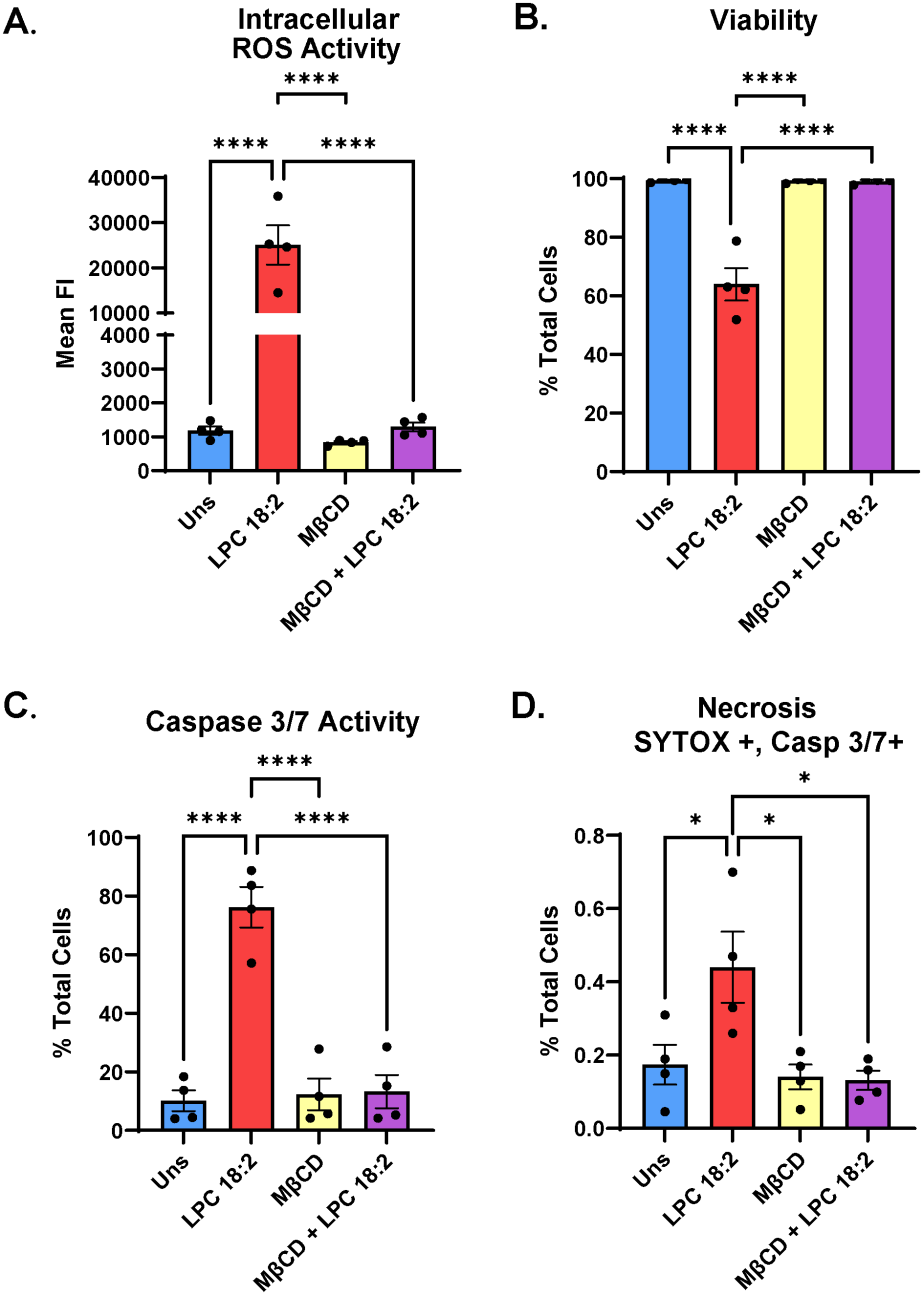
LPC 18:2-induced ROS and apoptosis are dependent on cholesterol and lipid rafts. **(A)** ROS induction in human neutrophils treated with medium alone (Uns), LPC 18:2 (30 μM), Methyl-β-cyclodextrin (MβCD) (1 mM), or LPC 18:2 (30 μM) and MβCD (1 mM) for 45 minutes following 35 minute MβCD pretreatment. **(B)** Viability (DAPI-%) of human neutrophils after treatment with medium alone (Uns), LPC 18:2 (30 μM), MβCD (1 mM), or LPC 18:2 (30 μM) and MβCD (1 mM) for 45 minutes following 35 minute MβCD pretreatment. **(C)** Human neutrophil apoptosis (cleaved caspase-3/7) after treatment with medium alone (Uns), LPC 18:2 (30 μM), MβCD (1 mM), or LPC 18:2 (30 μM) and MβCD (1 mM) for 45 minutes following 35 minute MβCD pretreatment. **(D)** Human neutrophil necrosis (SYTOX+ caspase-3/7 dual stain) after treatment with medium alone (Uns), LPC 18:2 (30 μM), MβCD (1 mM), or LPC 18:2 (30 μM) and MβCD (1 mM) for 45 minutes following 35 minute MβCD pretreatment. Statistics: Ordinary one-way ANOVA with Tukey’s test **(A–D)**; in all panels, bars = mean, error = SEM. (*p ≤ 0.05, **p ≤ 0.01, ***p ≤ 0.001, ****p ≤ 0.0001).

Lipid rafts are cholesterol– and sphingolipid–enriched membrane microdomains that function as organizing platforms for immune receptor signaling and lipid–protein interactions (39). Saturated LPC species, including LPC 16:0, have been shown to incorporate into lipid rafts and signal through these domains to regulate calcium flux and NADPH oxidase activity in leukocytes (40, 41). To determine whether LPC 18:2 signaling similarly depends on lipid raft integrity, primary human neutrophils were pretreated with methyl-β-cyclodextrin (MβCD) to deplete membrane cholesterol and disrupt lipid raft organization (42). Disruption of lipid rafts completely abolished LPC 18:2–induced intracellular reactive oxygen species (ROS) generation assessed by DHR and prevented the loss of viability measured by DAPI uptake (**Figure 4A, B**). MβCD pretreatment likewise eliminated LPC 18:2–mediated caspase-3/7 activation (**Figure 4C**) and abrogated cell death as defined by SYTOX/caspase-3/7^+^ staining (**Figure 4D**). These findings indicate that both oxidative stress induction and apoptotic signaling downstream of LPC 18:2 require intact lipid raft organization. Collectively, these results identify lipid rafts as essential scaffolds for LPC 18:2 engagement in neutrophils and suggest that this LPC species signals through lipid raft–dependent surface or membrane-proximal pathways.

## 4 Discussion

In this study, we identify LPC 18:2 as a bioactive lipid mediator that shapes human neutrophil fate and function in a manner distinct from the saturated LPC species LPC 16:0 *in vitro*. Upon treatment with physiologically relevant concentrations of LPC species, LPC 16:0 triggered inflammatory transcriptional and functional neutrophil phenotypes accompanied by membrane rupture and NETosis-like, lytic cell death. In contrast, LPC 18:2 promoted a transcriptionally quiescent, non-lytic apoptotic program characterized by intracellular ROS release and mitochondrial dysfunction. Mechanistically, intracellular ROS and caspase-3/7 activity induced by LPC 18:2 were dependent on cholesterol-rich lipid raft organization, implicating membrane microdomain architecture as a critical determinant of LPC 18:2 signaling. Thus, while both saturated and unsaturated LPC species influence neutrophil fate and function, LPC 16:0 appears to drive inflammatory neutrophil activation whereas LPC 18:2 preferentially promotes apoptotic clearance pathways that are less inflammatory. These divergent responses may help explain the opposing associations of LPC 16:0 and LPC 18:2 with inflammatory disease outcomes observed in both experimental and clinical settings *in vivo*.

Divergent phenotypes were reflected across multiple functional readouts. LPC 16:0 induced LDH and HMGB1 release, MPO activity, NE release, accumulation of CitH3, together with increased SYTOX uptake, indicating membrane rupture and a lytic, pro-inflammatory form of cell death resembling NETosis. The accumulation of extracellular CitH3 under non-permeabilizing staining conditions suggests chromatin decondensation and release into the extracellular space, a hallmark feature of NET formation and consistent with the broader loss of membrane integrity observed following LPC 16:0 stimulation. At the transcriptional level, LPC 16:0 robustly activated proinflammatory transcriptional programs, including immediate-early genes (*FOS, FOSB*) and inflammatory cytokines such as *CXCL8*.

In contrast to LPC 16:0, LPC 18:2 treatment of neutrophils markedly increased intracellular ROS generation and caspase-3/7 activation accompanied by mitochondrial membrane depolarization and cytochrome *c* release, but without widespread membrane rupture or NETosis-associated activity. Notably, LPC 18:2 produced more ROS than LPC 16:0 at equivalent conditions, yet caused less lytic death and failed to induce significant CitH3 externalization. Together, the absence of extracellular CitH3 and minimal evidence of membrane rupture support a comparatively non-lytic and transcriptionally quiescent death program consistent with activation of the intrinsic apoptosis pathway.

Our findings reinforce previous research demonstrating acyl chain–specific effects of distinct LPC species on neutrophils *in vitro*. LPC species are well recognized to exert chain-dependent immunological effects, with saturated LPCs generally promoting inflammation and polyunsaturated LPCs exhibiting non-inflammatory or inflammation-resolving properties (19, 27, 43, 44). Prior work by Ojala et al. demonstrated that in primary human neutrophils treated *in vitro* with LPCs, unsaturated LPC species induce a stronger respiratory burst than saturated species, which instead enhanced calcium influx and induced alterations in membrane permeability (27). Consistent with these observations, we find that LPC 18:2 elicits rapid and robust ROS production in neutrophils, particularly when compared to LPC 16:0. Extending these findings, we further demonstrate that ROS induction by LPC 18:2 occurs in the absence of membrane rupture or broad inflammatory transcription, instead engaging intrinsic apoptotic pathways. Our observed upregulation of *CXCL8* by LPC 16:0 but not LPC 18:2 is consistent with prior observations in human endothelial cells, where LPC 16:0, but not LPC 18:2, promoted *CXCL8* expression (45). Similarly, our finding that LPC 16:0, but not LPC 18:2, increased *PTGS2* (COX2) transcription aligns with studies showing selective upregulation of *PTGS2* by LPC 16:0 in endothelial cells (46). Notably, while Brkić et al. observed that both LPC species increased COX2 protein abundance, transcriptional induction was specific to LPC 16:0.

LPC 18:2 induced robust ROS production, mitochondrial dysfunction, cytochrome *c* release, and caspase-3/7 activation despite producing minimal transcriptional changes at an early 45-minute or later 2-hour timepoint. This observation is consistent with the unique biology of neutrophils, which execute rapid effector functions through transcription-independent mechanisms involving pre-existing signaling proteins, granule contents, metabolic pathways, and post-translational signaling networks. For example, neutrophil oxidative burst is mediated through rapid assembly and activation of the NADPH oxidase complex, while degranulation and chemotactic responses rely largely on preformed effector machinery rather than *de novo* gene expression (47, 48). Similarly, NET formation and other neutrophil death programs can proceed despite pharmacologic inhibition of transcription or translation, highlighting the importance of signaling events downstream of calcium flux, kinase activation, and chromatin remodeling (49). In this context, the absence of a broad transcriptional signature following LPC 18:2 exposure should not be interpreted as a lack of biological activity. Rather, our findings suggest that LPC 18:2 primarily engages rapid signaling pathways that alter neutrophil fate and function before substantial transcriptional reprogramming occurs. The emergence of a limited IFIT-associated signature after 2 hours further supports the possibility that transcriptional responses to LPC 18:2 are delayed and secondary to earlier signaling events. This later signature may alternatively represent a transcriptional response to the functional phenotype LPC 18:2 induces. The interferon-induced IFIT family signature could reflect a cellular response to mislocated nucleic acids or other molecules released during LPC 18:2-induced apoptosis.

Building on these observations, we propose that the LPC 18:2 response represents a two-phase pro-resolving program, in which transient ROS production may support antimicrobial or tumoricidal functions, followed by commitment to intrinsic apoptosis that enables controlled resolution. Importantly, our mitochondrial studies indicate that mitochondrial activation and mitochondrial ROS production are not synonymous in LPC 18:2-treated neutrophils (**Figure 3**). LPC 18:2 induced a modest (non-significant) increase in mitochondrial superoxide that was abolished by MitoTEMPO, supporting the presence of mitochondrial ROS generation. However, MitoTEMPO had little effect on total intracellular ROS levels in the same samples, indicating that mitochondria are not the dominant source of the observed robust ROS response elicited by LPC 18:2 (**Figure 3E, F**). The precise origin of the non-mitochondrial ROS remains to be defined. Dedicated NOX2-directed studies including PMA-stimulated positive controls or genetic NOX2 inhibition will be needed to resolve this mechanism. These findings are nevertheless compatible with our MitoTracker results (**Figure 3C, D**), which revealed increased fluorescence intensity within the remaining highly polarized mitochondrial population, suggesting enhanced mitochondrial activity in a subset of viable neutrophils. Together, these observations support a model in which LPC 18:2 promotes mitochondrial remodeling and intrinsic apoptotic signaling while simultaneously inducing substantial ROS production from sources that remain to be fully defined.

Mechanistically, our data demonstrate that LPC 18:2–induced ROS production and apoptosis require intact lipid rafts, cholesterol-rich membrane microdomains that organize signaling complexes. Disruption of lipid rafts through cholesterol depletion completely abrogated LPC 18:2–induced signals, indicating that membrane organization is essential for LPC 18:2 signaling in human neutrophils. This requirement for intact lipid rafts is consistent with prior studies showing raft-dependent signaling by saturated LPC species, including LPC 16:0, in other cellular contexts (40, 41, 50, 51). To our knowledge, this represents the first evidence that unsaturated LPC species likewise depend on lipid raft integrity for signal transduction. These findings suggest that LPC 18:2 may either engage a raft-resident receptor or directly incorporate into membrane microdomains to initiate downstream signaling. Additional studies will be required to define the precise molecular intermediates linking raft-associated signaling to mitochondrial apoptosis. This work provides an initial mechanistic framework for understanding how LPC 18:2 may confer protection against severe ICB-irAEs and other inflammatory events by reprogramming neutrophil responses.

*In vivo*, apoptotic neutrophils are efficiently cleared by macrophages through efferocytosis, a process that actively promotes anti-inflammatory and tissue-reparative programs mediated by TGF-β, IL-10, and specialized pro-resolving lipid mediators (52, 53). Given that neutrophil infiltration and epithelial damage are prominent features of ICB-induced colitis (53), LPC 18:2–driven apoptotic remodeling of neutrophil responses could potentially limit mucosal injury by facilitating macrophage-mediated resolution rather than sustained inflammation. Thus, ROS-concurrent intrinsic apoptosis may represent a therapeutic equilibrium that restrains ICB-irAE–associated inflammation while preserving the anti-tumor immune competence required for effective immune checkpoint blockade. The effects of LPC 18:2 on neutrophil survival are also likely to be influenced by the inflammatory milieu. Neutrophils exposed to priming signals such as GM-CSF, TNF, microbial products, and endothelial-derived mediators undergo substantial functional reprogramming, including altered activation thresholds and enhanced survival through suppression of constitutive apoptotic pathways (54). As a result, the pro-apoptotic phenotype observed following LPC 18:2 exposure in resting neutrophils may differ in activated or tissue-resident neutrophils that integrate multiple inflammatory cues simultaneously. Defining how inflammatory priming shapes LPC 18:2 signaling will therefore be an important area for future investigation and may provide additional insight into its immunomodulatory effects *in vivo* (55).

Significantly, LPC 18:2 displays more biochemical versatility than LPC 16:0. LPC 16:0 is a poor substrate for further enzymatic modification, whereas unsaturated LPC 18:2 can be oxidized or remodeled by lipoxygenases, cytochrome P450 enzymes, and acyltransferases, generating bioactive derivatives such as 9- and 13-hydroxyoctadecadienoic acids (HODEs) or re-entering phosphatidylcholine pools via LPCAT-mediated reacylation (56–59). These downstream metabolites participate in anti-inflammatory signaling, PPARδ activation, and membrane lipid remodeling, features largely absent from saturated LPC species (60–64). Thus, LPC 18:2 possesses greater biochemical “versatility” to engage antioxidant and inflammation-resolving pathways. An additional consideration is that LPC 18:2 may undergo further metabolic remodeling after cellular exposure. Extracellular LPC species can be converted by autotaxin (ENPP2) into lysophosphatidic acid (LPA), while intracellular LPC can be reacylated by lysophosphatidylcholine acyltransferases (LPCATs) to regenerate phosphatidylcholine as part of the Lands cycle (65–68). These pathways represent major metabolic fates of unsaturated LPC species and may influence the biological availability and persistence of LPC 18:2 in inflammatory tissues. Despite this turnover, circulating LPC levels remain orders of magnitude higher than LPA (which are typically ~30–60 nM), indicating that LPC species are not rapidly depleted under physiological conditions *in vivo* (19, 38). Thus, while local bioavailable concentrations of LPCs at the neutrophil membrane *in vivo* are difficult to directly measure, and the functional thresholds for LPCs to induce apoptosis *in vivo* are not determined, bulk plasma LPC concentrations support the physiological plausibility of the concentration range used in our study.

Although our study did not directly quantify LPC 18:2 metabolites, the absence of broad transcriptional changes despite robust ROS production and apoptotic signaling suggests that any conversion through autotaxin- or LPCAT-dependent pathways does not result in substantial transcriptional activation of neutrophils within the experimental time frame examined. Additionally, the lack of a significant increase in caspase 3/7 activity after treatment with LPA 18:2 suggests that LPA 18:2 does not drive the functional phenotypes observed in this study. Instead, our findings support a model in which LPC 18:2 primarily exerts rapid membrane-proximal and post-translational effects, while its subsequent metabolic remodeling may contribute to longer-term regulation of lipid signaling and membrane composition. Future isotope-tracing and lipidomic studies will be required to define the relative contribution of these pathways to neutrophil fate decisions.

This study has several limitations. All experiments were performed *in vitro* using isolated primary human neutrophils, which may not fully recapitulate the complex signaling environment present *in vivo*. In circulation, LPCs are largely bound to albumin (63), a factor not examined here that may influence LPC bioavailability and signaling. In addition, while we focused on the two most abundant plasma LPC species, other LPC species may exert distinct or context-dependent effects on neutrophils and other immune cell populations. Finally, although our data establish a cholesterol- and lipid raft–dependent mechanism, the downstream molecular mediators remain undefined. Addressing these gaps will be essential to establish the physiological and therapeutic relevance of LPC–neutrophil interactions.

In summary, our findings identify LPC 18:2 as a transcriptionally quiescent lipid mediator that promotes neutrophil apoptosis through cholesterol- and lipid raft–dependent signaling. These results highlight the functional diversity of lysophospholipids and underscore their capacity to shape immune cell fate. More broadly, this work suggests that selective modulation of LPC species may represent a novel therapeutic strategy for restraining pathological neutrophil activation in inflammatory settings, including immune checkpoint blockade–associated toxicities.

## Data Availability

The datasets presented in this study can be found in online repositories. The names of the repository/repositories and accession number(s) can be found below: GSE311051 and GSE336476 (GEO).

## Supplementary material

Supplementary Figure 1 Flow cytometry gating strategies. **(A)** Gating strategy for human neutrophil cleaved caspase-3/7 assay. **(B)** Gating strategy for human neutrophil ROS assay. **(C)** Gating strategy for human neutrophil citrullinated Histone H3 (CitH3) assay. **(D)** Gating strategy for human neutrophil cytochrome c release assay. **(E)** Gating strategy for human neutrophil MitoTracker assay. **(F)** Gating strategy for human neutrophil MitoSOX assay.

Supplementary Figure 2 LPC 18:2 induces a limited interferon-induced IFIT-family gene program after 2 hour treatment. **(A)** Principal component analysis (PCA) of human neutrophils (n = 3) treated with medium alone (Uns) or LPC 18:2 (10 μM) for 2 hours. **(B)** Volcano plot of differentially expressed protein-coding genes (DEGs) between LPC 18:2-treated neutrophils and unstimulated cells (DESeq2). DEG significance threshold: FDR ≤ 0.01, |log₂FC| > 0.5, mean TPM > 10. **(C)** Top 10 significantly upregulated neutrophil-related genes after LPC 18:2 treatment. **(D)** Top 9 significantly downregulated neutrophil-related genes after LPC 18:2 treatment.
